# CLIP4 Shows Putative Tumor Suppressor Characteristics in Breast Cancer: An Integrated Analysis

**DOI:** 10.3389/fmolb.2020.616190

**Published:** 2021-01-26

**Authors:** Yu Fan, Lijia He, Yu Wang, Shaozhi Fu, Yunwei Han, Juan Fan, Qinglian Wen

**Affiliations:** ^1^ Department of Oncology, The Affiliated Hospital of Southwest Medical University, Nuclear Medicine and Molecular Imaging Key Laboratory of Sichuan Province, Academician (Expert) Workstation of Sichuan Province, Luzhou, China; ^2^ Health Management Department, The Affiliated Hospital of Southwest Medical University, Luzhou, China

**Keywords:** DNA methylation, CAP-Gly domain containing linker protein family member 4, breast cancer, prognosis, biomarker, integrated analysis

## Abstract

**Background**: CAP-Gly domain containing linker protein family member 4 (CLIP4) plays an important role in cancers. However, its expression, prognostic value, and biological effect in breast cancer remain unclear.

**Methods**: Data on patients diagnosed with breast cancer were retrieved from the TCGA-BRCA and other public omics databases. The expression profile of CLIP4 was analyzed using Oncomine, bc-GenExMiner, and TCGA. The prognostic value of CLIP4 was determined by Kaplan-Meier Plotter and Human Protein Atlas. Identification of genes co-expressed with CLIP4 and potential mechanism analyses were performed using UALCAN, STRING, Metascape, and GSEA. The epigenetic characteristics of CLIP4 were determined by DiseaseMeth and MEXPRESS.

**Results**: CLIP4 was downregulated and its expression was negatively correlated with estrogen receptor (ER), progesterone receptor (PR), human epidermal growth factor receptor type 2 (HER2) status, Nottingham prognostic index (NPI), and Scarff-Bloom-Richardson (SBR) grade in breast cancer, whereas it was positively linked to basal-like and triple negative breast cancer status. Ectopic expression of CLIP4 was related with poor prognosis. In the analysis of genes co-expressed with CLIP4, GSEA showed that the Hedgehog (Hh), JAK-STAT, ERBB, Wnt signaling pathway, cell adhesion molecules, and pathways in cancer were dissimilarly enriched in the CLIP4 expression high phenotype. Analysis of the genetics and epigenetics of CLIP4 indicated that its expression was negatively correlated with DNA methylation.

**Conclusion**: Methylated CLIP4 may be a novel prognostic and therapeutic biomarker for breast cancer.

## Introduction

Breast cancer is the most common female malignancy in China and is the main cause of mortality in Western countries (Chen et al., 2016; Siegel et al., 2018). Moreover, the incidence of breast cancer is gradually rising in most countries (DeSantis et al., 2019). Despite advances in early screening, diagnosis, and treatment of breast cancer, the overall prognosis for patients remains poor. Thus, it is urgent to find sensitive and specific biomarkers for breast cancer.

Microtubules have a dynamic structure that continuously changes during growth and shrinkage (Mitchison and Kirschner, 1984). Microtubule-associated proteins (MAPs) influence microtubule properties. One group of MAPs, the plus end binding proteins (or +TIPs), bind to and stabilize microtubule plus ends. Mammalian cytoplasmic linker protein (CLIP)-170, links microtubule plus ends to kinomeres, endocytosis vesicles, and the leading edge of migrating cells (Howard and Hyman, 2003), is a prototypical +TIP (Perez et al., 1999). The CLIP-170 family (CLIP1, CLIP2, CLIP3, and CLIP4) associates microtubules with cellular organelles through a cytoskeleton-associated protein glycine rich (CAP-Gly) domain. The CAP-Gly domain, which is conserved among organisms, is a small 80-residue protein module (Riehemann and Sorg, 1993). CAP-Gly domains are important to the function of CLIPs and many other proteins, and are implicated in cell polarity maintenance, intracellular transport, cell migration, and oncogenesis (Galjart and Perez, 2003; Akhmanova and Hoogenraad, 2005; Galjart, 2005). Recent research on CLIP4 uncovered its potential functions in cancers. However, the role of CLIP4 in breast cancer remains unknown.

Here, we first evaluated the expression profile of CLIP4 in breast cancer by data mining. The prognostic value of CLIP4 was also analyzed. To gain further insight into the molecular mechanisms involved in breast cancer-related CLIP4 regulatory networks, GSEA was used. Finally, the regulation of the expression of CLIP4 in breast cancer was investigated by genetic and epigenetic analyses.

## Methods

### Expression profile analysis

The expression pattern of CLIP4 was analyzed using Oncomine and TCGA. First, the expression profile of CLIP4 was analyzed with Oncomine, which facilitates investigation of cancer microarray databases and genome-wide expression analysis (Rhodes et al., 2004). The cut-off of p-value, fold change, and gene rank were defined as 0.05, 2, and 10% (Fan et al., 2019), respectively. The gene expression (1,098 cases) and corresponding clinical data were downloaded from TCGA official website for Breast Invasive Carcinoma (TCGA-BRCA). All statistical analyses were performed using R (v.3.6.3). The association with clinical features and CLIP4 was analyzed with the Wilcoxon signed-rank and logistic regression test.

bc-GenExMiner (Jezequel et al., 2012) was used to assess relationships among CLIP4 expression and clinicopathological features including age, nodal status, hormone receptor status (ER and PR), HER2, pathological subtype, NPI, and SBR grade. A value of *p* <0.05 was statistically significant.

### Survival analysis

The Kaplan-Meier Plotter (http://kmplot.com/analysis/), an online tool established from gene expression and survival data of cancer patients originated from the GEO database (Gyorffy et al., 2010), was used to evaluate the prognostic value of CLIP4 in breast cancer. The survival plot, hazards ratio (HR), 95% confidence interval (CI), and log-rank p were displayed on the web page. The prognostic significance are available from Human Protein Atlas (https://www.proteinatlas.org/) (Uhlen et al., 2005). A log-rank *p* < 0.05 was statistically significant.

### Analysis of Co-Expression and Protein-Protein Interaction Networks

The genes co-expressed with CLIP4 were analyzed using UALCAN (Chandrashekar et al., 2017). A total of 687 genes positively and negatively correlated with CLIP4 in breast cancer were downloaded. Genes with a Pearson’s correlation coefficient ≥0.3 (absolute value) were included. The protein-protein interaction network was established using STRING v11.0 and was based on co-expressed genes (https://string-db.org/cgi/input.pl/) (Szklarczyk et al., 2019). The confidence score was defined as 0.4 (Fan et al., 2019).

### Metascape and Gene Set Enrichment Analysis

Gene ontology (GO) and pathway enrichment analysis of CLIP4-associated genes were performed using Metascape (http://metascape.org/) (Zhou et al., 2019). A computational method GSEA was used to determine whether a prior defined set of genes shows statistically significant, concordant differences between two biological states (Subramanian et al., 2005). In this study, an ordered list of genes was first generated by GSEA based on correlation with CLIP4 expression. The significant survival difference observed between high and low CLIP4 was elucidated. Gene set permutations were performed 1,000 times each analysis. The expression level of CLIP4 was used as a phenotype label. The nominal p-value and normalized enrichment score (NES) were used to classify the pathways enriched in each phenotype.

### Analysis of DNA Methylation and Genetic Alterations

To further investigate the regulatory effect of DNA methylation modification on CLIP4 mRNA expression, we used DiseaseMeth, version 2.0, which was updated along with the increased DNA methylation data and associations between diseases and genes. These datasets were collected from revolutionary large international disease projects such as TCGA and GEO among others (Xiong et al., 2017). Then, 871 breast invasive carcinoma samples in MEXPRESS (https://mexpress.be/) (Koch et al., 2015) datasets were analyzed to evaluate the CLIP4 gene. The genetic alterations of CLIP4 were identified by a web portal (http://www.cbioportal.org/) (Cerami et al., 2012) evaluated from breast invasive carcinoma (TCGA, Firehose Legacy, 1108 samples).

## Results

### The Expression of CLIP4 in Breast Cancer

**FIGURE 1:**
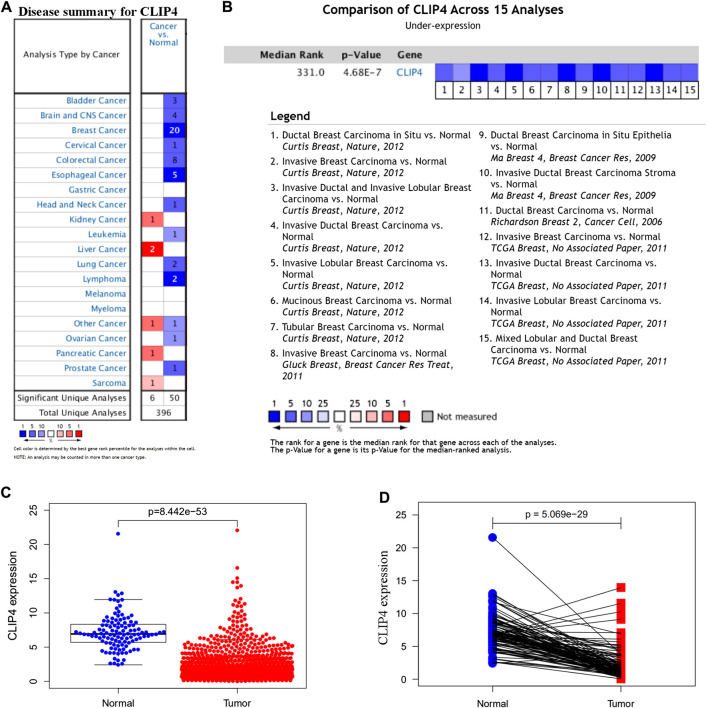
Expression pattern of CLIP4 in breast cancer. **(A)** Expression of CLIP4 in different human cancers by analysis of cancer and normal tissue based on Oncomine; **(B)** Oncomine meta-analysis for the expression of CLIP4 in breast cancer; **(C)** CLIP4 mRNA expression in breast cancer and normal tissue (TCGA datasets); and **(D)** CLIP4 mRNA expression in breast cancer and adjacent normal tissue (TCGA datasets).

To identify the roles of CLIP4 in cancers, we searched the Oncomine dataset for CLIP4 mRNA expression in common cancer types. Comparison of cancer and normal samples was performed to analyze the expression pattern of CLIP4 in breast cancer (Figure 1A). The meta-analysis based on Oncomine was used to evaluate the integrated and median expression of CLIP4 across 15 analyses (*p* = 4.68E−7, <0.001) (Figure 1B). As shown in Table 1, the CLIP4 expression was significantly decreased in invasive breast carcinoma, invasive lobular breast carcinoma, invasive ductal breast carcinoma, tubular breast carcinoma, mucinous breast carcinoma, ductal breast carcinoma *in situ*, and mixed lobular and ductal breast carcinoma (Richardson et al., 2006; Ma et al., 2009; Curtis et al., 2012; Gluck et al., 2012). For further validation, we also investigated the expression of CLIP4 from TCGA datasets. As shown in Figure 1C, CLIP4 expression was dramatically lower in breast cancer than in normal tissues (*p* = 8.44E−53, <0.001). Furthermore, the paired plot for CLIP4 showed that the expression in breast cancer was significantly downregulated compared with adjacent normal samples (*p* = 5.07E−29, <0.001) (Figure 1D). Taken together, the combination of bioinformatic analyses demonstrated that CLIP4 was significantly downregulated in breast cancer.

**TABLE 1:** The significant changes of CLIP4 in breast cancer from oncomine datasets.

Types of cancer vs normal tissues	Fold change (>2)	*t*-test	*p*-value (<1E-4)	Reporter ID
Invasive ductal breast carcinoma	−2.268	−33.329	4.48E−79	ILMN_1759792
Invasive lobular breast carcinoma	−2.215	−23.245	2.12E−68	ILMN_1759792
Invasive ductal and invasive lobular breast carcinoma	−2.253	−19.951	8.72E−47	ILMN_1759792
Tubular breast carcinoma	−2.130	−16.210	2.42E−31	ILMN_1759792
Mucinous breast carcinoma	−2.187	−14.080	9.84E−22	ILMN_1759792
Ductal breast carcinoma in situ	−2.187	−9.924	4.68E−7	ILMN_1759792
Invasive breast carcinoma	−2.158	−6.366	1.15E−6	ILMN_1759792
Invasive ductal breast carcinoma	−5.492	−27.864	4.28E−70	A_23_P209230
Invasive breast carcinoma	−3.581	−12.411	1.09E−22	A_23_P209230
Invasive lobular breast carcinoma	−3.466	−11.370	1.22E−15	A_23_P209230
Mixed lobular and ductal breast carcinoma	−2.242	−7.247	1.15E−5	A_23_P209230
Invasive breast carcinoma	−3.053	−14.220	4.71E−9	27167
Ductal breast carcinoma	−3.054	−5.849	3.36E−7	226425_at
Invasive ductal breast carcinoma stroma	−2.615	−5.451	4.71E−5	Hs.56123.0.S1_3p_at
Ductal breast carcinoma in situ epithelia	−5.191	−5.361	9.44E−5	

**FIGURE 2:**
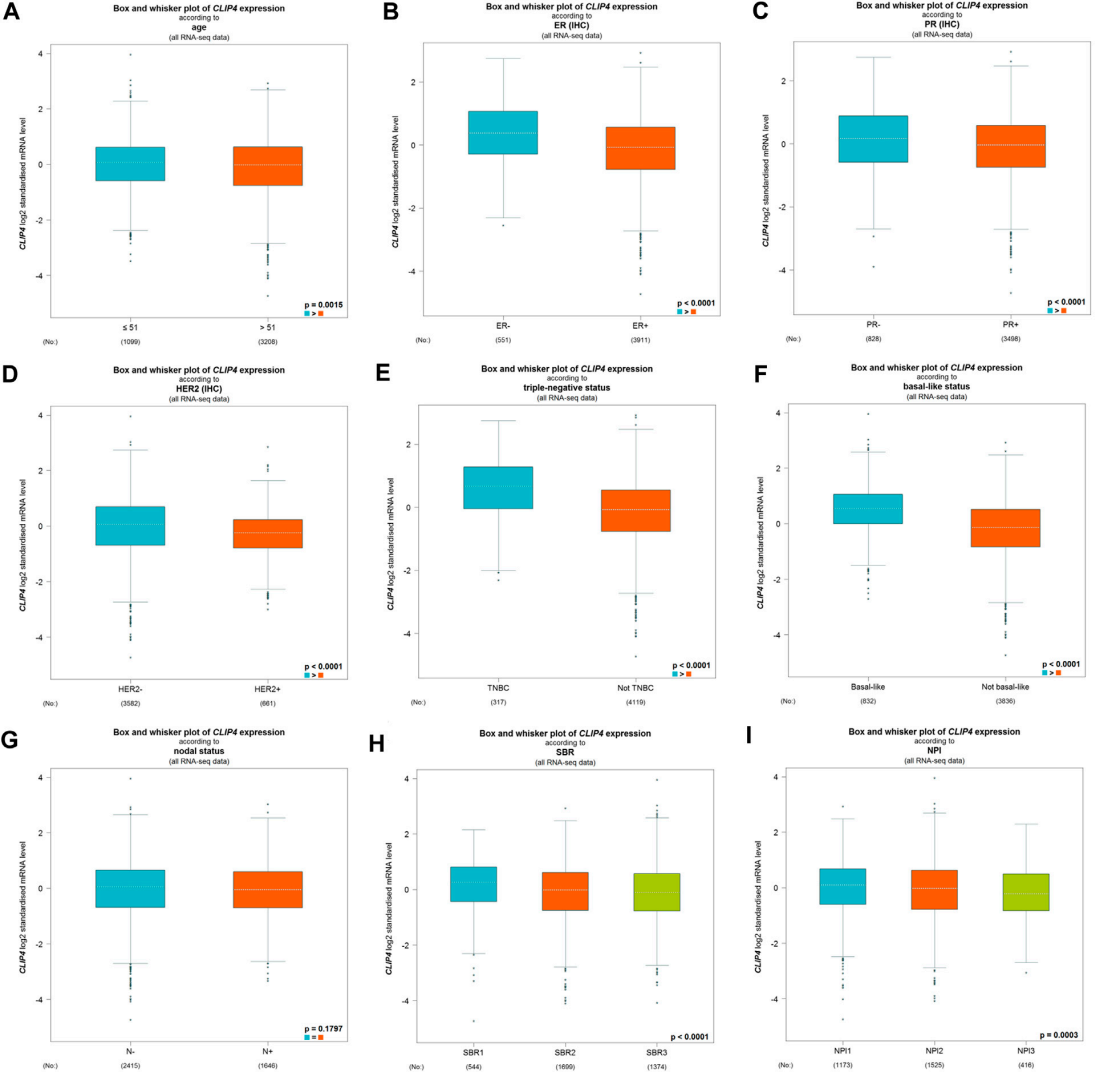
Relationships among CLIP4 expression and clinicopathological features of breast cancer based on the bc-GenExMiner database. **(A)** Box plot for the association of CLIP4 mRNA expression and age; **(B)** box plot for the association of CLIP4 mRNA expression and ER; **(C)** box plot for the association of CLIP4 mRNA expression and PR; **(D)** box plot for the association of CLIP4 mRNA expression and HER2; **(E)** box plot for the association of CLIP4 mRNA expression and triple-negative status; **(F)** box plot for the association of CLIP4 mRNA expression and basal-like status; **(G)** box plot for the association of CLIP4 mRNA expression and nodal status; **(H)** box plot for the association of CLIP4 mRNA expression and SBR; and **(I)** box plot for the association of CLIP4 mRNA expression and NPI.

We further evaluated the associations between CLIP4 expression and clinicopathological characteristics using the bc-GenExMiner tool (Figure 2). There was no significant difference of CLIP4 expression between the nodal-positive and -negative groups (*p* = 0.1797, >0.05). Age, ER, PR, HER2 status, NPI, and SBR grade were negatively related to CLIP4 expression (Age: *p* = 0.0015, <0.01; ER status: *p* < 0.0001; PR status: *p* < 0.0001; HER2 status: *p* < 0.0001; NPI: *p* = 0.0003, <0.001; SBR grade: *p* < 0.0001). However, the expression of CLIP4 was markedly upregulated in basal-like and triple-negative breast cancer (TNBC) (basal-like status: *p* < 0.0001; triple-negative status: *p* < 0.0001). To further evaluate the association between TNM stage and CLIP4 expression, we performed analysis using R (v.3.6.3) based on TCGA datasets. The results showed that the T, N, and stage were negatively associated with CLIP4 expression (T3-4 vs. T1-2: *p* = 0.008, <0.01; nodal-positive vs. -negative: *p* = 0.046, <0.05; stage III-IV vs. stage I-II: p = 0.027, <0.05) (Supplementary Figure S1). However, logistic regression analysis showed no significant differences between T, N, stage and CLIP4 expression (*p* > 0.05) (Supplementary Table S1 **)**. In short, these results indicated that CLIP4 expression may play a favorable role in breast cancer patients.

### Prognostic Significance of CLIP4 in Breast Cancer

**FIGURE 3:**
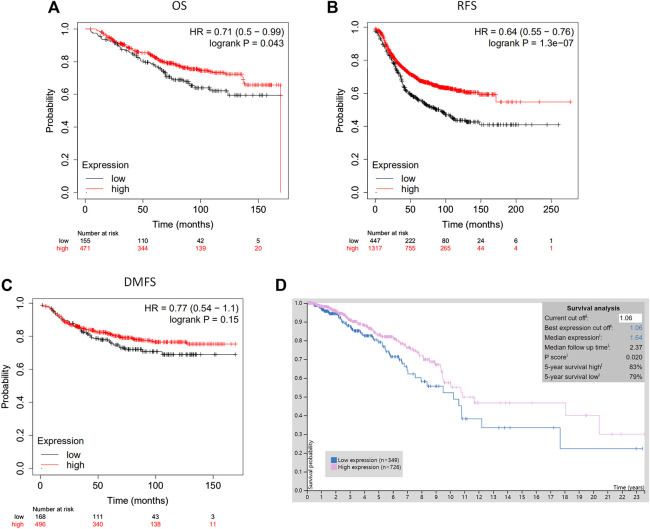
The prognostic significance of CLIP4 for breast cancer determined by Kaplan-Meier Plotter and TCGA datasets. **(A)** High CLIP4 expression shows a better OS for breast cancer; **(B)** high CLIP4 expression shows a better RFS for breast cancer; **(C)** high CLIP4 expression shows a better DMFS for breast cancer; **(D)** the 5-year survival rate for patients with breast cancer (*n* = 726) with greater CLIP4 mRNA expression (83%) better for patients (*n* = 349) than low expression (79%) (TCGA datasets).

The analysis using the Kaplan-Meier plotter based on 626 breast cancer patients showed that CLIP4 upregulation was significantly associated with better overall survival (OS) [HR: 0.71 (0.50–0.99); *p* = 0.043, <0.05, Figure 3A]. High expression of CLIP4 was associated with better relapse-free survival (RFS) in 1,764 breast cancer patients [HR: 0.64 (0.55–0.76); *p* = 1.30E-07, Figure 3B]. Furthermore, CLIP4 was also positively associated with distant metastasis-free survival (DMFS) in breast cancer, although statistical significance was not reached [HR: 0.77 (0.54–1.10); *p* = 0.150, >0.05, Figure 3C]. Based on TCGA dataset and the optimal cutoff value, the survival rate was significantly better in breast cancer patients (*n* = 726) with high expression of CLIP4 than in those with low expression (*n* = 349) (*p* = 0.020, <0.05) (https://www.proteinatlas.org/) (Figure 3D). In summary, CLIP4 may be a novel prognostic indicator in breast cancer patients. Meanwhile, the value of CLIP4 in different intrinsic subtypes of breast cancer was evaluated using the Kaplan-Meier plotter. High CLIP4 expression in three intrinsic subtypes (Luminal A, Luminal B, and HER2 positive) but not in the basal-like subtype was correlated with better OS [Luminal A: HR: 0.63 (0.37–1.07), *p* = 0.085; Luminal B: HR: 0.46 (0.21–1.01), *p* = 0.047; HER2 positive: HR: 0.46 (0.21–1.02), *p* = 0.050] (Supplementary Figure S2).

### Genes Co-Expressed with CLIP4 and Potential Biomolecular Networks

**FIGURE 4:**
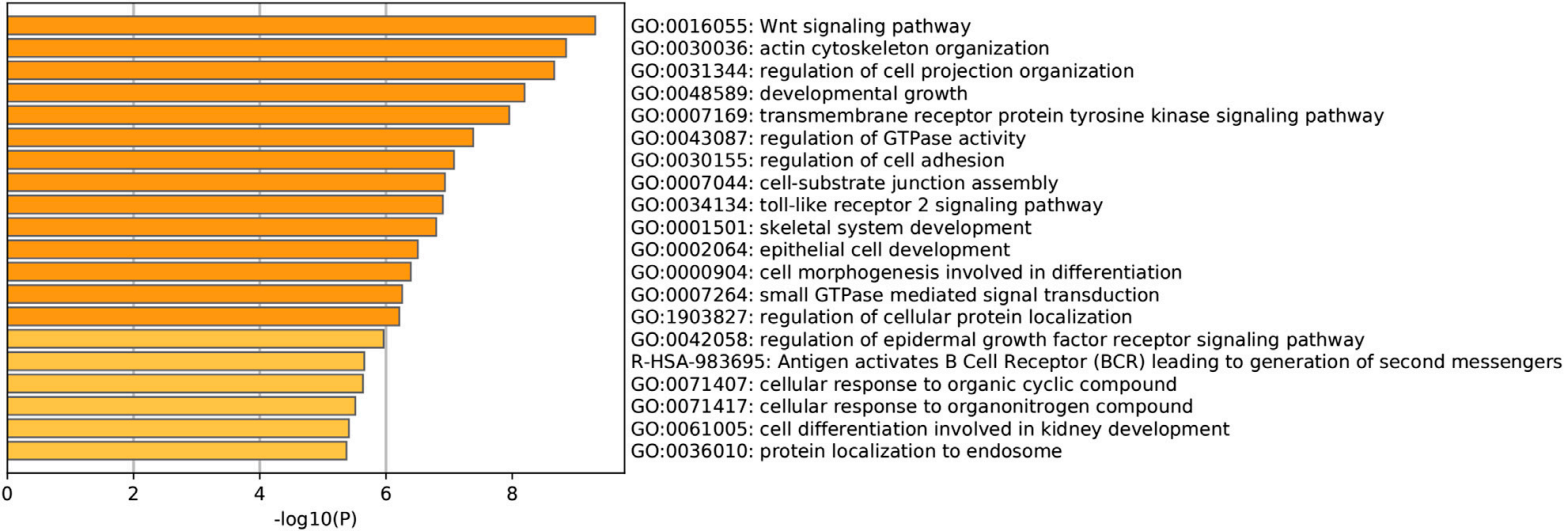
Pathway enrichment analysis of CLIP4 co-expressed genes. The top 20 pathway enrichment clusters based on Metascape analysis of CLIP4 co-expressed genes carried out with GO Biological Processes and Reactome Gene Sets. Length of bars represent log_10_ (*p*-value) determined by the best-scoring term within each cluster.

**FIGURE 5:**
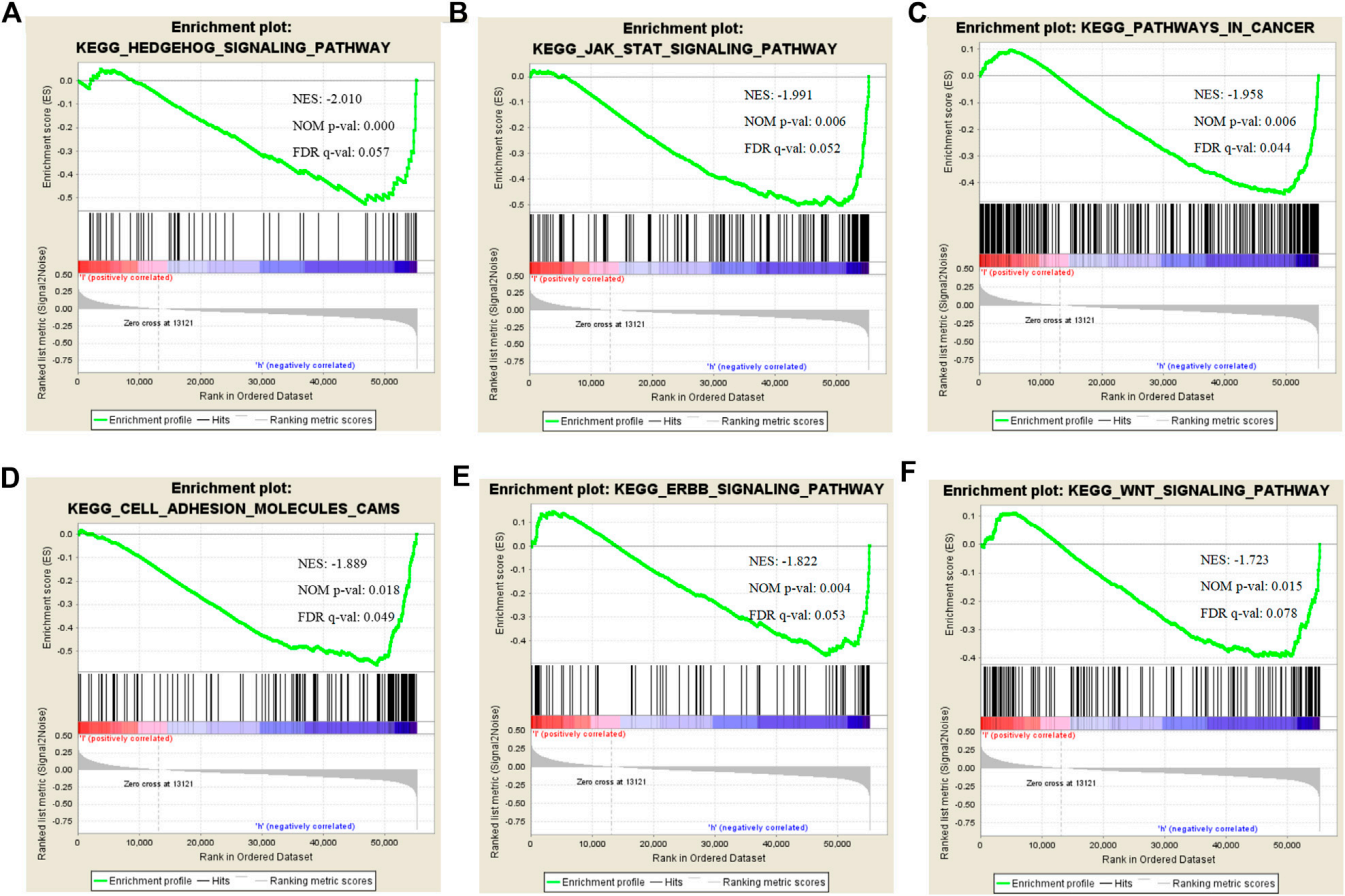
Enrichment plots for CLIP4 in breast cancer obtained by GSEA. GSEA results showing Hedgehog signaling pathway **(A)**, JAK-STAT signaling pathway **(B)**, pathways in cancer **(C)**, cell adhesion molecules **(D)**, ERBB signaling pathway **(E)**, and Wnt signaling pathway **(F)** are differentially enriched in CLIP4-related breast cancer. NES, normalized ES; NOM *p*-value, normalized *p*-value; FDR *q*-value, false discovery rate *q*-value.

To find the potential role and regulatory mechanism of CLIP4 in breast cancer, genes co-expressed with CLIP4 were predicted using the UALCAN database, and 687 genes were identified (Supplementary Table S2 **)**. A PPI network for CLIP4 co-expressed genes, based on experimental evidence, was constructed using the STRING database (Supplementary Figure S3). We further performed GO and pathway enrichment analysis for these genes using Metascape (Figure 4). The top 20 clusters with their enriched terms, which originated from two categories, were included: GO biological process (BP) and Reactome gene sets. The representative enriched GO functions for these co-expressed genes included Wnt signaling pathway (GO: 0016055), actin cytoskeleton organization (GO: 0030036), developmental growth (GO: 0048589), transmembrane receptor protein tyrosine kinase signaling pathway (GO: 0007169), regulation of GTPase activity (GO: 0043087), toll-like receptor 2 signaling pathway (GO: 0034134), and regulation of epidermal growth factor receptor signaling pathway (GO: 0042058). To further identify molecular signaling pathways differentially activated in breast cancer, GSEA between CLIP4 low and high expression datasets was performed (Supplementary Table S3, S4). GSEA identified significant differences (FDR *q*-value <0.25, NOM *p*-value <0.05) in enrichment of the MSigDB Collection (c2.cp.kegg.v6.2.symbols.gmt). The most enriched tumor-associated signaling pathways were selected based on their NES values (Wu and Zhang, 2018) (Figure 5; Table 2). As shown in Figure 5, Hh signaling pathway, JAK-STAT signaling pathway, cell adhesion molecules, ERBB signaling pathway, Wnt signaling pathway, and pathways in cancer were differentially enriched in the CLIP4-high expression phenotype. These results demonstrated that upregulation of CLIP4 in breast cancer may involve the Wnt, ERBB, or other tumor-associated signaling pathways. Another result based on multiple GSEA analysis is shown in Supplementary Figure S4.

**TABLE 2:** The most enriched and tumor-associated signaling pathways in phenotype high.

MSigDB collection	Gene set name	ES	NES	NOM *p*-value	FDR *q*-value
c2.cp.kegg.v6.2.symbols.gmt	HEDGEHOG_SIGNALING_PATHWAY	−0.528	−2.010	0.000	0.057
	JAK_STAT_SIGNALING_PATHWAY	−0.504	−1.991	0.006	0.052
	PATHWAYS_IN_CANCER	−0.442	−1.958	0.006	0.044
	CELL_ADHESION_MOLECULES_CAMS	−0.561	−1.889	0.018	0.049
	ERBB_SIGNALING_PATHWAY	−0.463	−1.822	0.004	0.053
	WNT_SIGNALING_PATHWAY	−0.396	−1.723	0.015	0.077

Note: NES, normalized enrichment score; NOM, nominal; FDR, false discovery rate. Gene sets with NOM *p*-val < 0.05 and FDR *q*-val < 0.25 are considered as significant.

### Promoter Methylation of CLIP4 in Breast Cancer

**FIGURE 6:**
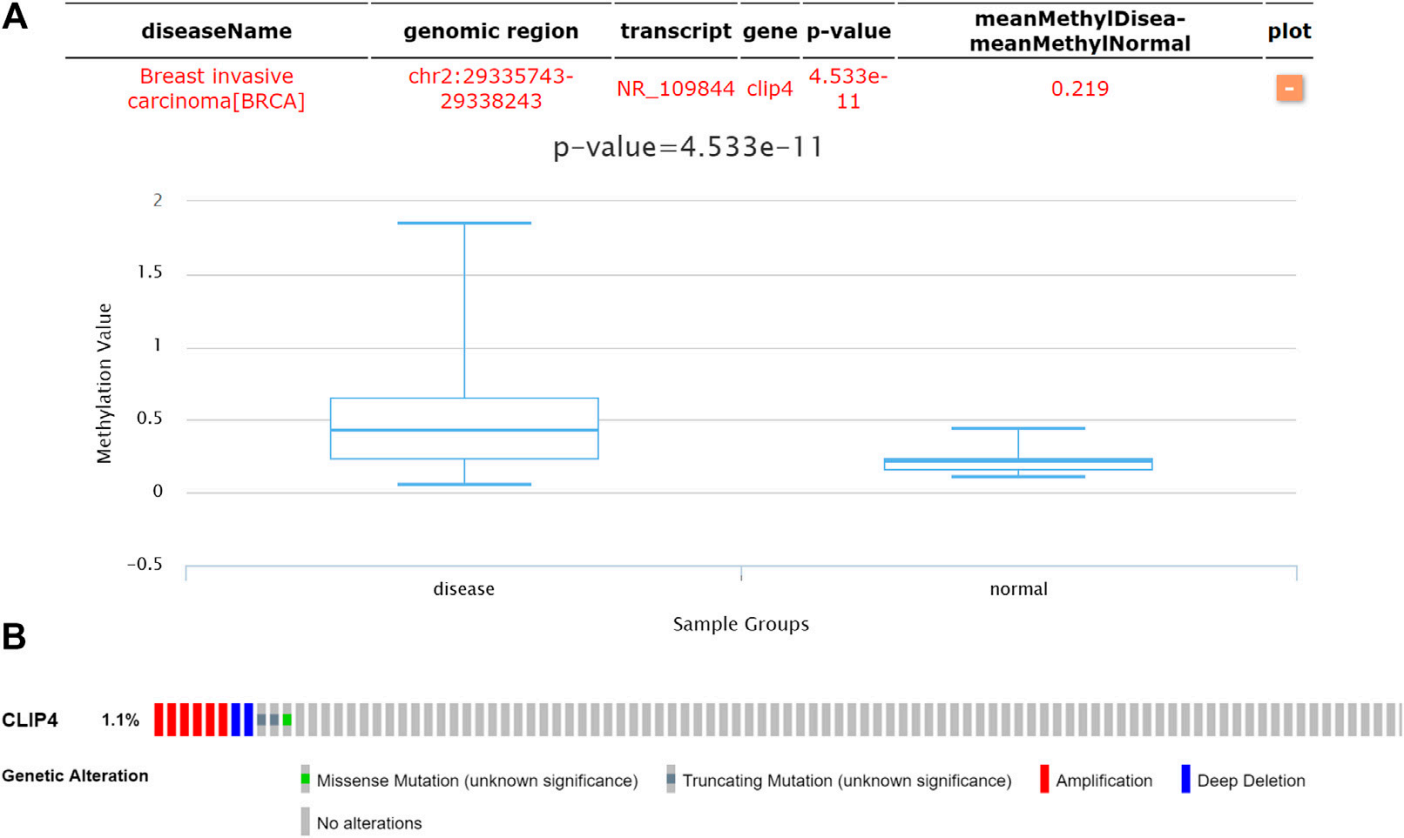
Genetic features of CLIP4 in breast cancer. **(A)** The DNA methylation status of CLIP4 in 1299 breast invasive carcinomas and in normal tissue obtained from DiseaseMeth datasets. **(B)** CLIP4 gene alterations in 963 breast cancers based on OncoPrint. Tumor tissues are shown in columns.

To assess whether CLIP4 downregulation was related with DNA methylation in breast cancer, the DiseaseMeth database was analyzed. The results showed that the CLIP4 promoter was hypermethylated in patients compared with normal controls (*p* = 4.533E−11, <0.001) (Figure 6A). MEXPRESS was used to confirm the promoter methylation status of CLIP4 in breast cancer. The samples were presented in ascending order according to the level of expression, and CLIP4 expression was negatively associated with DNA methylation based on Pearson’s correlation analyses (Supplementary Figure S5). The results indicated that promoter hypermethylation of CLIP4 was related to the downregulation of mRNA expression. Furthermore, we investigated whether CLIP4 downregulation was caused by genetic alterations. Copy number alterations and gene mutations of CLIP4 were analyzed using the cBioPortal online tool. Genetic alterations of CLIP4 were mutually exclusive and only found in 11 (1.1%) of 963 invasive breast carcinoma patients, of which six samples had DNA amplification, two had deep deletion, two had a truncating mutation, and one had a missense mutations (Figure 6B). These results supported the significant role of DNA methylation in the regulation of CLIP4 expression.

## Discussion

In the present study, the expression of CLIP4 was investigated in breast cancer by bioinformatics analysis. Oncomine and TCGA analysis revealed mRNA expression of CLIP4 to be significantly decreased in breast cancer, when compared to normal samples. CLIP4 expression was negatively correlated with HER2 status, NPI, SBR grade, nodal status, and tumor stage. However, the basal-like and TNBC types were positively associated with CLIP4 expression. The prognostic role of CLIP4 in breast cancer was then investigated using the Kaplan-Meier Plotter, and the results demonstrated that higher expression of CLIP4 was associated with better RFS and OS, especially in Luminal B and HER2 positive breast cancers. Analysis of epigenetic and genetic alterations of CLIP4 in breast cancer indicated that promoter methylation was the main mechanism underlying the regulation of CLIP4 gene expression. These findings suggested that promoter methylation-mediated loss of CLIP4 expression may be a novel prognostic biomarker for breast cancer.

Cytoskeletal proteins play important effects in cellular functions such as opposing compression, invasion, migration, and transport. The cytoskeleton also provides an optimal scaffold for the process of signal transduction (Balikov et al., 2017). However, the role of the cytoskeleton and microtubule-associated protein CLIP4 in human cancer has not been studied extensively. The methylation of XKR6, CCDC57, MAML3, SDC2, and CLIP4 is associated with age and tumor location in gastric cancer (Chong et al., 2014). The promoter methylation of IRF4, ELMO1, CLIP4, and MSC is related with increasing seriousness from gastritis with no metaplasia to gastritis with metaplasia and gastric cancer (Pirini et al., 2017). Contrary to the results of this study, increased expression of four genes (CLIP4, NOX4, LAMP5, and MATN3) is related to poor prognosis, and the expression of these genes is higher in stromal components than in epithelial cancer cells in gastric cancer (Lee et al., 2014). CLIP4 shows high expression levels in kidney cancer cell lines compared with normal cell lines, and CLIP4 significantly increases cell migration and viability (Ahn et al., 2016). In clear cell renal cell carcinoma (ccRCC), CLIP4 mutations are three-fold higher in patients with aggressive tumors than in those without aggressive ccRCC, and high expression levels of MOCOS, BAIAP2L1, DDX11, and CLIP4 are markedly associated with poor OS (Park et al., 2020). Interestingly, potentially pathogenic mutations of CLIP4 and other genes may state a subset of lung adenocarcinoma among never-smoking women (Donner et al., 2018). In line with this study, 43 genes (including CLIP4) were downregulated in ovarian cancer tumor samples and reactivated after treatment with 5-Aza-2'-deoxycytidine (5-aza-dC); CLIP4, GULP1, BAMBI, NT5E, and TGFB2 showed a pattern of cancer-specific methylation (Maldonado et al., 2018). Three DNA methylation markers, C9orf50, KCNQ5, and CLIP4, can discriminate between the plasma from colorectal cancer patients and that of healthy individuals (Jensen et al., 2019). Furthermore, CLIP4 has been considered a promising epigenetic biomarker by analysis of differentially methylated genes and differentially expressed genes between matched tumor and non-tumor tissues of the colon (Wu et al., 2020). Of note, CLIP4 was associated with better OS in Luminal B and HER2 positive breast cancers but not TNBC type in this study, the result revealed that CLIP4 may be used as a target to overcome the drug resistance, incomplete responders, and relapsed in individualized treatment of Luminal B and HER2 breast cancer. Despite many studies providing important data and tumor-specific roles for CLIP4 in human cancer, its biological function and molecular mechanism remain unclear. Another CAP-Gly domain containing linker protein, CLIP1 is a mRNA stemness index-related key gene associated with a better lung adenocarcinoma prognosis, which is involved in tumor metastasis, relapse, and drug resistance (Zhao et al., 2020). A report showed that cells expressing FGFR2-CLIP1 fusion were sensitive to INCB054828 (a pan-FGFR inhibitor) in cholangiocarcinoma while the FGFR2 N549H mutation was resistant to this inhibitor (Krook et al., 2019).

Unlike previous reports, analyzing gastric cancer and ccRCC, this study showed that high CLIP4 expression was associated with a better prognosis, suggesting that CLIP4 had a suppressive function in breast cancer. To elucidate the molecular mechanism underlying the function of CLIP4 in breast cancer, we constructed a CLIP4-associated regulatory network. The present findings supported the important role of CLIP4 upregulation in tumor-associated signaling pathways such as Hh, JAK-STAT, Wnt, and ERBB, and suggested that it acted as a tumor suppressor in breast cancer. GSEA and Metascape analysis indicated that CLIP4 was related to the Wnt signaling pathway. Wnt signaling plays a critical role in normal development as well as tumorigenesis (Yin et al., 2018). Overactivation of Wnt signaling is implicated in human diseases including breast cancer. However, the association of CLIP4 with Wnt signaling and cytoskeletal proteins in human cancers has not been reported to date. Tankyrases, which are multifunctional poly (ADP-ribose) polymerase (PARP) superfamily members with features of both signaling and cytoskeletal proteins, antagonize the Wnt/β-catenin signaling (Kuusela et al., 2016). In addition, the Wnt/β-catenin pathway can influence the distribution of microtubules and neurofilaments (Tian et al., 2019). As a therapeutic target to overcome drug resistance in breast cancer (Tabassum et al., 2019), the JAK-STAT signaling pathway was associated with CLIP4 in this study. However, only one previous study has shown that another cytoskeletal protein, CLIP3, plays an essential role in astrocyte activation, and is associated with STAT3 pathway activation induced by spinal cord injury (Chen et al., 2018). Therefore, there is lack of other evidence in human cancers that identifies the role of CLIPs in JAK-STAT signaling. Similarly, one study reported that the cytoskeletal protein Zyxin is involved in fine tuning the neural plate patterning in *Xenopus laevis* embryos by modulating the activity of an effector of Hh signaling, the transcription factor glioma-associated oncogene 1 (GLI1) (Martynova et al., 2018). Hh signaling, which results in activation of GLI transcription factors and correlates with worse outcomes of breast cancer, is activated in human mammary stem cells (Bhateja et al., 2019). Although therapeutic agents such as Herceptin, which are designed to inhibit ERBB activity, have dramatically improved the survival of patients with HER2 positive breast cancer, approximately 50% of patients acquire drug resistance within 1 year (Zahnow, 2006). Therefore, it is important to identify gene targets associated with ERBB signaling. The present findings on the association between CLIP4 and ERBB signaling in breast cancer may provide a novel research direction.

In summary, the cytoplasmic linker protein CLIP4 may act as a novel prognostic and epigenetic biomarker for breast cancer patients. There were limitations to this study. First, the analysis was based on mRNA levels from public datasets, which requires confirmation at the mRNA and protein level. Second, some of the bioinformatics tools used had only limited functionality, such as hierarchical analysis without multivariable Cox regression analysis. Finally, there was a lack of in vivo and in vitro direct evidence to confirm the biological function and molecular mechanism of CLIP4 in breast cancer. Additional basic studies and clinical trials are urgently needed to validate the findings of this study. For the future, we plan to confirm and evaluate the value of CLIP4 as a potential biomarker for breast cancer.

## Data Availability Statement

The datasets presented in this study can be found in online repositories. The names of the repository/repositories and accession number(s) can be found in the article/Supplementary Material.

## Author Contributions

Study deign: YF and LH; investigation and resources: YF; data collection: YF, SF, YH, and JF; writing-original draft preparation: YF and YW; writing-review and editing: YF and YW; data interpretation and visualization: LH and QW; supervision: YF; project administration: YF; funding acquisition: YF; statistical analysis: LH, YW, and SF. All authors have read and agreed to the published version of the manuscript.

## Funding

This work was supported by the Funded Project of Affiliated Hospital of Southwest Medical University for Doctors (grant no. 17135), and the Luzhou-Southwest Medical University Applied Basic Research Project (grant no. 2019LZXNYDJ07).

## Conflict of Interest

The authors declare that the research was conducted in the absence of any commercial or financial relationships that could be construed as a potential conflict of interest.
