# Supplementary material for: CLIP4 Shows Putative Tumor Suppressor Characteristics in Breast Cancer: An Integrated Analysis
Source: Front Mol Biosci. 2021 Jan 26;7:616190. doi: 10.3389/fmolb.2020.616190 (PMC7870488; doi:10.3389/fmolb.2020.616190)
Supplement: Supplementary file 1 [file table1.docx]

**Table S1 The associations between CLIP4 expression and clinicopathological characteristics (logistic regression analysis)**

| **Clinical characteristic** | **N**  **(total)** | **Odds ratio**  **(OR)** | **95% Confidence interval**  **(95%CI)** | **P-value** |
| --- | --- | --- | --- | --- |
| T(1-2 vs. 3-4) | 1074 | 0.761 | 0.549-1.052 | 0.100 |
| N (0 vs. N1-3) | 1057 | 0.818 | 0.642-1.041 | 0.103 |
| Stage (I-II vs. III-IV) | 1054 | 0.776 | 0.586-1.026 | 0.076 |
