# Supplementary material for: CLIP4 Shows Putative Tumor Suppressor Characteristics in Breast Cancer: An Integrated Analysis
Source: Front Mol Biosci. 2021 Jan 26;7:616190. doi: 10.3389/fmolb.2020.616190 (PMC7870488; doi:10.3389/fmolb.2020.616190)
Supplement: Supplementary file 2 [file table2.docx]

**Table S2 Co-expressed genes of CLIP4 obtained by UALCAN database**

| Co-expressed genes of CLIP4 (including up and down-regulated genes) |
| --- |
| GLS |
| CCDC82 |
| LY75 |
| MAML2 |
| PPARA |
| ZC3H12C |
| NCK1 |
| EHBP1 |
| PRNP |
| FAM126A |
| ANXA1 |
| RBMS1 |
| TLR1 |
| ARHGAP21 |
| PELI1 |
| FOXN2 |
| QKI |
| RASAL2 |
| CLCN4 |
| TRIM2 |
| RHOQ |
| SOCS5 |
| UBXN2A |
| CHST3 |
| SOS1 |
| NAB1 |
| ITGB8 |
| IFNAR2 |
| PTPN14 |
| ADAM17 |
| FNDC3B |
| JRKL |
| TLR6 |
| LPCAT2 |
| MOBKL2B |
| RIMS3 |
| RDX |
| FAM49A |
| NFAT5 |
| TLE4 |
| LYN |
| C22orf23 |
| PPP1CB |
| ATL2 |
| FAM3C |
| DIAPH2 |
| ARAP2 |
| RNF150 |
| PAPSS1 |
| TANK |
| NCK2 |
| TTC7A |
| OSBPL3 |
| CTTNBP2NL |
| CCDC50 |
| FZD7 |
| PLEKHG1 |
| RPS6KA3 |
| USP6NL |
| PLEKHM3 |
| FAM135A |
| ACSL4 |
| ADPRH |
| ROCK2 |
| ANXA3 |
| BIRC2 |
| EVC2 |
| LOC100302401 |
| TMBIM1 |
| STON1 |
| PLS3 |
| MYO1E |
| MICALL1 |
| ARNTL2 |
| PM20D2 |
| PRKD3 |
| CPNE8 |
| ARHGAP31 |
| MSN |
| RIOK3 |
| BAG2 |
| UBASH3B |
| WDR43 |
| CDK6 |
| CBL |
| RBM7 |
| ESYT2 |
| FAS |
| MID1 |
| RAB12 |
| ADCY7 |
| MPZL2 |
| TIGD2 |
| TMEM43 |
| KCTD9 |
| UBE2E3 |
| MAP7D3 |
| FMNL2 |
| MDFIC |
| LATS2 |
| SERPINB8 |
| ZNF532 |
| MBP |
| RRAS2 |
| USP31 |
| UGP2 |
| SIRPA |
| HCG11 |
| LRRK1 |
| DAPP1 |
| SVIL |
| TIAM2 |
| TES |
| DSG3 |
| C7orf60 |
| PTAR1 |
| PLCG2 |
| WWTR1 |
| MTMR2 |
| YAP1 |
| EML4 |
| DOCK7 |
| AKT3 |
| MSL3 |
| ITSN2 |
| SH3KBP1 |
| GTDC1 |
| KIAA1804 |
| PROS1 |
| CLIC4 |
| APLF |
| CCDC88A |
| CMPK1 |
| LUZP1 |
| CSDAP1 |
| CFLAR |
| HSPC159 |
| C10orf25 |
| ANKRD6 |
| MRAS |
| RASGEF1B |
| NMT2 |
| OTUD4 |
| ANKRD36BP1 |
| SMC5 |
| RUNX3 |
| KLHL21 |
| BICD2 |
| ROR1 |
| FAM171A1 |
| NCKAP1 |
| CREB3L2 |
| GPR125 |
| TMEM117 |
| L3MBTL4 |
| PRKX |
| JOSD1 |
| NPC1 |
| LAMB3 |
| CD47 |
| CYP7B1 |
| ABI1 |
| KLF5 |
| AEBP2 |
| PPTC7 |
| PRICKLE1 |
| FRMD3 |
| PKP1 |
| RGMA |
| KIF1B |
| ARL4C |
| NT5DC3 |
| TBC1D1 |
| MCFD2 |
| CDC14B |
| VPS54 |
| SMURF1 |
| RASA2 |
| GPN1 |
| GLIPR1 |
| ZNF462 |
| TRIM29 |
| TRDMT1 |
| LSM14A |
| OPTN |
| CCNY |
| TTLL4 |
| MBNL1 |
| MAP2 |
| PTPLA |
| TMEM200A |
| OBFC2A |
| ITPRIPL1 |
| KCNK5 |
| AGTPBP1 |
| RRAGC |
| MPP6 |
| STK38 |
| ZNF507 |
| RAPGEF2 |
| LRRC8C |
| TGFA |
| PAPD7 |
| KIRREL |
| SLC39A14 |
| C3orf64 |
| PLAGL1 |
| ZFR |
| DSE |
| DSG2 |
| CLCN5 |
| ATP11C |
| FAM105B |
| FGD4 |
| SLC9A6 |
| RNF217 |
| RCN1 |
| FOXC1 |
| ANKS6 |
| CXCL16 |
| FUT4 |
| GABRP |
| GPR75 |
| RAB23 |
| IFRD1 |
| CDKL1 |
| LIMS1 |
| OSBPL9 |
| DPYSL2 |
| NFIL3 |
| ALDH1L2 |
| TRPC1 |
| FNDC4 |
| FAT1 |
| ARL5B |
| COMMD2 |
| AMOTL1 |
| HPS5 |
| MEX3C |
| CMTM7 |
| IL18R1 |
| YEATS2 |
| BNIP2 |
| C15orf41 |
| PAQR3 |
| ANKH |
| CLASP1 |
| LOC100190939 |
| EIF5A2 |
| KLF12 |
| PABPC4L |
| GPM6B |
| EFNA5 |
| CELF2 |
| C9orf85 |
| FAM168B |
| RND3 |
| ASAM |
| BACH1 |
| PTPN4 |
| IFFO2 |
| DSC2 |
| RBMS2 |
| LOC151162 |
| C21orf91 |
| TCF7L2 |
| MFHAS1 |
| PATL1 |
| CORO1C |
| KCMF1 |
| INPP4A |
| SERPINB5 |
| TMEM20 |
| SACS |
| KHDRBS3 |
| OGFRL1 |
| CWC22 |
| CAV2 |
| DSC3 |
| PITPNB |
| SMCHD1 |
| IGF2BP2 |
| LRP6 |
| USP34 |
| PROM1 |
| TMEM30A |
| LRP12 |
| CALD1 |
| CRYAB |
| IL34 |
| CSDA |
| ST5 |
| TMEM123 |
| LRIG3 |
| ZNF644 |
| TULP3 |
| C13orf18 |
| SCHIP1 |
| PLSCR4 |
| CFL2 |
| ADORA2B |
| SERAC1 |
| SFRP1 |
| RBM9 |
| EPS15 |
| KLF11 |
| TMEM194B |
| LIMK2 |
| CALU |
| TMEM71 |
| ETS1 |
| LOC387647 |
| SPTBN1 |
| DPYD |
| C9orf21 |
| SFT2D2 |
| ACTN1 |
| RIF1 |
| MLLT10 |
| TMEM39A |
| MLXIP |
| EIF2C4 |
| PLXNA1 |
| C9orf30 |
| SNCA |
| FOXN3 |
| LBR |
| MALT1 |
| RCAN3 |
| FZD6 |
| HMGN4 |
| KDM5A |
| INO80D |
| IFI16 |
| PPL |
| TSPAN2 |
| BTN2A2 |
| KLF13 |
| IQCG |
| ETV6 |
| FAM160A1 |
| AHR |
| PPFIBP1 |
| ZNF567 |
| SEPT10 |
| RIPK2 |
| ZNF280C |
| TMEM170A |
| ASAP1 |
| EXT1 |
| C2orf56 |
| LPIN1 |
| RLF |
| GPR180 |
| CARD6 |
| CCDC93 |
| EPB41L2 |
| H6PD |
| TJP2 |
| SAMD4A |
| DDX18 |
| BICD1 |
| XRN1 |
| TCF20 |
| C11orf41 |
| SAV1 |
| ST3GAL6 |
| PIKFYVE |
| DARS |
| PRRG1 |
| DISC1 |
| FAM57A |
| LOC400927 |
| IL1RAP |
| APOBEC3C |
| LOC285033 |
| PAQR8 |
| GLG1 |
| TMOD2 |
| MYO9A |
| SH3BP2 |
| LOC399959 |
| ITGB4 |
| GTPBP8 |
| NOTCH1 |
| CEP170 |
| KLRAQ1 |
| HNRPLL |
| ANAPC1 |
| CDH3 |
| VEPH1 |
| GAN |
| SLC25A32 |
| LEPROT |
| SGTB |
| FAM123B |
| PGM2 |
| KIAA0754 |
| STRN |
| HS2ST1 |
| DNAJB4 |
| CRLF3 |
| EPC2 |
| STARD7 |
| TAF4B |
| RAPGEF1 |
| DDX26B |
| ZNF267 |
| RHBDF2 |
| DDR2 |
| SMEK2 |
| PIK3AP1 |
| LAMP2 |
| ASXL1 |
| TSPAN33 |
| PLEKHA1 |
| PIWIL4 |
| LHFPL2 |
| LUZP6 |
| GPRIN2 |
| PAPOLG |
| C3orf55 |
| RYK |
| SH3GLB1 |
| QPCT |
| ANKRD27 |
| APP |
| C1RL |
| ENOX2 |
| RARB |
| FAM126B |
| SSPN |
| MEF2A |
| RAI14 |
| SIRPB1 |
| ZDHHC18 |
| TMEM220 |
| GRAMD3 |
| SOD2 |
| ARHGEF9 |
| RSU1 |
| SYNM |
| BIRC3 |
| MIA |
| SPAST |
| C1S |
| HDAC4 |
| SPSB1 |
| BACH2 |
| ERC1 |
| TFCP2L1 |
| TMEM217 |
| PIM1 |
| SCPEP1 |
| TEAD1 |
| CHST11 |
| SGK269 |
| MAFG |
| NDEL1 |
| YIPF4 |
| ZRANB2 |
| DUSP11 |
| KPNA3 |
| KIAA0355 |
| FOXL1 |
| YES1 |
| TLR2 |
| PKD2 |
| MGAT5 |
| ARL10 |
| MAP3K2 |
| GNPTAB |
| FBXO48 |
| KIAA0947 |
| ALS2CR4 |
| SLFN11 |
| RNF19B |
| BTBD3 |
| TUBB6 |
| PPM1F |
| ADAMTS5 |
| EIF2C3 |
| TEX10 |
| CHMP4C |
| ADARB1 |
| PHACTR2 |
| ZCCHC11 |
| LOC728819 |
| PTCH1 |
| VPS13A |
| TBC1D4 |
| WTAP |
| OSBPL11 |
| SLFN12 |
| NFE2L2 |
| ADCY3 |
| SDHD |
| C2orf67 |
| ZHX1 |
| TTC33 |
| KANK1 |
| RAB42 |
| ATP11A |
| PRKAG2 |
| METTL4 |
| SERBP1 |
| CEP57 |
| ARL6IP6 |
| PICALM |
| MYLK |
| EMP1 |
| ZNF521 |
| CAD |
| BICC1 |
| TMEM17 |
| DZIP1 |
| GPR161 |
| LRCH1 |
| ACTR3 |
| RAB7L1 |
| PSAT1 |
| SPRY3 |
| RHBDL2 |
| UBA2 |
| NHSL1 |
| ITGA9 |
| BACE2 |
| ANXA8L2 |
| SOX10 |
| TCF7L1 |
| PLSCR1 |
| ATP10D |
| SLC16A1 |
| NRP2 |
| HSPA4L |
| CHIC2 |
| SOX9 |
| CSNK2A2 |
| TMEM38A |
| OSMR |
| PRTFDC1 |
| SLC12A6 |
| TBX19 |
| FAM107A |
| SUPT7L |
| ABI2 |
| ULBP3 |
| ZNF518B |
| NCRNA00120 |
| ABHD13 |
| DCBLD1 |
| HAPLN3 |
| CACHD1 |
| CCNYL1 |
| PNRC1 |
| ZFP82 |
| ZNF292 |
| BTC |
| PLXDC2 |
| NFIB |
| KIAA1826 |
| NT5C2 |
| AKAP13 |
| MMP7 |
| ZNF146 |
| PCNP |
| SCLT1 |
| MTHFD1L |
| KDM4D |
| PAG1 |
| ATP2C1 |
| MAPK14 |
| INPP1 |
| ATG9A |
| ATP6V1E2 |
| PLEKHG6 |
| MEMO1 |
| MAPK1 |
| STARD4 |
| ANKRD13A |
| AGPAT9 |
| CA13 |
| EPHA2 |
| FBXO17 |
| FAM60A |
| FERMT2 |
| RASSF2 |
| MICAL3 |
| MPZL3 |
| SRPK1 |
| STK24 |
| POLH |
| ECHDC1 |
| TXNRD3IT1 |
| C6orf204 |
| HIVEP2 |
| DOCK5 |
| SLFN5 |
| PCNXL2 |
| ARHGEF10 |
| KRT23 |
| MMGT1 |
| SMO |
| TRAM1 |
| LDHB |
| C1GALT1 |
| SIK2 |
| C1R |
| KIAA1755 |
| GABARAPL1 |
| GOPC |
| KIAA1671 |
| FGFR1OP2 |
| XPO5 |
| BBOX1 |
| SEL1L3 |
| ZNF750 |
| RPE |
| ETV3 |
| ZDHHC20 |
| NFE2L3 |
| TTL |
| RAP2A |
| TET1 |
| LOC283314 |
| PLEKHG2 |
| GABPA |
| ZNF512 |
| C12orf4 |
| SLC5A3 |
| TPST1 |
| NPAT |
| ATF7IP |
| EIF2C1 |
| CFI |
| ICAM1 |
| NPR2 |
| HIATL1 |
| AMD1 |
| SPRY2 |
| XPO4 |
| NSMAF |
| B3GNT5 |
| PCNX |
| C3orf17 |
| RIMKLB |
| BMPR2 |
| ATG4C |
| STK17B |
| PGCP |
| IPO5 |
| SIKE1 |
| ZFAND5 |
| C2orf3 |
| FAM108B1 |
| PPPDE1 |
| APOL6 |
| PTPRK |
| MFSD4 |
| NMI |
| ZNF383 |
| SPDEF |
| C15orf63 |
| NDUFA2 |
| WIBG |
| ATP5G2 |
| SERF2 |
| MRPL54 |
| CYB561D2 |
| C14orf179 |
| CREB3L4 |
| FAAH |
| SNRNP35 |
| DCXR |
| NDUFB10 |
| GAMT |
| UQCRQ |
| FLYWCH2 |
| MRPS34 |
| SNRNP25 |
| UQCR11 |
| WBP1 |
| C17orf28 |
| MRPL53 |
| TEX264 |
| C12orf10 |
| BLOC1S1 |
