# Supplementary material for: CLIP4 Shows Putative Tumor Suppressor Characteristics in Breast Cancer: An Integrated Analysis
Source: Front Mol Biosci. 2021 Jan 26;7:616190. doi: 10.3389/fmolb.2020.616190 (PMC7870488; doi:10.3389/fmolb.2020.616190)
Supplement: Supplementary file 3 [file table3.docx]

**Table S3 Gene sets enriched in phenotype low CLIP4 expression**

| **NAME** | **ES** | **NES** | **NOM p-val** | **FDR q-val** |
| --- | --- | --- | --- | --- |
| KEGG_OXIDATIVE_PHOSPHORYLATION | 0.7648363 | 2.2947 | 0 | 0.002228478 |
| KEGG_PROTEIN_EXPORT | 0.8122975 | 2.2798128 | 0 | 0.001617916 |
| KEGG_HUNTINGTONS_DISEASE | 0.61105025 | 2.2058477 | 0 | 0.002561543 |
| KEGG_PARKINSONS_DISEASE | 0.7158589 | 2.1998265 | 0 | 0.002357146 |
| KEGG_BASE_EXCISION_REPAIR | 0.72912073 | 2.0585322 | 0.002 | 0.009425061 |
| KEGG_PROTEASOME | 0.7662467 | 2.0372574 | 0.002057613 | 0.011178513 |
| KEGG_ALZHEIMERS_DISEASE | 0.5571274 | 2.0337906 | 0 | 0.009909848 |
| KEGG_PEROXISOME | 0.5868515 | 1.9731855 | 0 | 0.01696832 |
| KEGG_NUCLEOTIDE_EXCISION_REPAIR | 0.6216072 | 1.9520559 | 0.005813954 | 0.019816825 |
| KEGG_CITRATE_CYCLE_TCA_CYCLE | 0.6579193 | 1.8760266 | 0.011090573 | 0.036422443 |
| KEGG_GLYCOSYLPHOSPHATIDYLINOSITOL_GPI_ANCHOR_BIOSYNTHESIS | 0.6636301 | 1.8356111 | 0.021611001 | 0.046187144 |
| KEGG_TERPENOID_BACKBONE_BIOSYNTHESIS | 0.7054897 | 1.8285278 | 0.014522822 | 0.044750858 |
| KEGG_PORPHYRIN_AND_CHLOROPHYLL_METABOLISM | 0.5725591 | 1.7964553 | 0.013888889 | 0.054023884 |
| KEGG_GLUTATHIONE_METABOLISM | 0.5499591 | 1.7864128 | 0.015748031 | 0.05441649 |
| KEGG_OTHER_GLYCAN_DEGRADATION | 0.68465 | 1.7587773 | 0.009803922 | 0.0616106 |
| KEGG_AMINOACYL_TRNA_BIOSYNTHESIS | 0.6531151 | 1.737455 | 0.017475728 | 0.06659378 |
| KEGG_FRUCTOSE_AND_MANNOSE_METABOLISM | 0.5202857 | 1.7175208 | 0.019193858 | 0.07167709 |
| KEGG_PYRIMIDINE_METABOLISM | 0.46514553 | 1.701638 | 0.032128513 | 0.07480688 |
| KEGG_BIOSYNTHESIS_OF_UNSATURATED_FATTY_ACIDS | 0.59027576 | 1.6816001 | 0.021653544 | 0.08054854 |
| KEGG_STEROID_BIOSYNTHESIS | 0.63142335 | 1.6201715 | 0.055888224 | 0.109086394 |
| KEGG_N_GLYCAN_BIOSYNTHESIS | 0.4978826 | 1.5916486 | 0.04263566 | 0.12078325 |
| KEGG_VASOPRESSIN_REGULATED_WATER_REABSORPTION | 0.46149048 | 1.5865297 | 0.04255319 | 0.11881844 |
| KEGG_DNA_REPLICATION | 0.61814165 | 1.5793391 | 0.112426035 | 0.11799798 |
| KEGG_CARDIAC_MUSCLE_CONTRACTION | 0.47987172 | 1.5758488 | 0.03195489 | 0.11517067 |
| KEGG_LYSOSOME | 0.44983542 | 1.5603564 | 0.07905138 | 0.12007529 |
| KEGG_SPLICEOSOME | 0.48567814 | 1.5385182 | 0.114754096 | 0.12942454 |
| KEGG_BUTANOATE_METABOLISM | 0.46927565 | 1.5367639 | 0.049723756 | 0.12598287 |
| KEGG_ARGININE_AND_PROLINE_METABOLISM | 0.43155617 | 1.5224751 | 0.036821704 | 0.13004921 |
| KEGG_MISMATCH_REPAIR | 0.56806386 | 1.5089786 | 0.10789981 | 0.13463794 |
| KEGG_GLYOXYLATE_AND_DICARBOXYLATE_METABOLISM | 0.5357844 | 1.4787853 | 0.07378641 | 0.151002 |
| KEGG_VIBRIO_CHOLERAE_INFECTION | 0.4283054 | 1.4729114 | 0.07254902 | 0.15106834 |
| KEGG_VALINE_LEUCINE_AND_ISOLEUCINE_DEGRADATION | 0.4661503 | 1.468987 | 0.09318996 | 0.14896083 |
| KEGG_AMINO_SUGAR_AND_NUCLEOTIDE_SUGAR_METABOLISM | 0.44146013 | 1.4560102 | 0.08514851 | 0.15325771 |
| KEGG_PYRUVATE_METABOLISM | 0.41264933 | 1.4153543 | 0.08253359 | 0.1803643 |
| KEGG_PANTOTHENATE_AND_COA_BIOSYNTHESIS | 0.46566644 | 1.3497342 | 0.14395393 | 0.23410366 |
| KEGG_PURINE_METABOLISM | 0.30732176 | 1.2879364 | 0.14901961 | 0.29366294 |
| KEGG_SNARE_INTERACTIONS_IN_VESICULAR_TRANSPORT | 0.37977162 | 1.2775271 | 0.17729084 | 0.2987793 |
| KEGG_RIBOFLAVIN_METABOLISM | 0.4031813 | 1.2345726 | 0.19560878 | 0.34149712 |
| KEGG_RNA_POLYMERASE | 0.4138425 | 1.2326752 | 0.24015749 | 0.33469 |
| KEGG_SELENOAMINO_ACID_METABOLISM | 0.40001944 | 1.2282695 | 0.22011386 | 0.33197156 |
| KEGG_PROPANOATE_METABOLISM | 0.38775852 | 1.1921903 | 0.25698325 | 0.36816025 |
| KEGG_PHENYLALANINE_METABOLISM | 0.42520744 | 1.1725982 | 0.26112187 | 0.38599467 |
| KEGG_ALANINE_ASPARTATE_AND_GLUTAMATE_METABOLISM | 0.34785882 | 1.1691742 | 0.23552124 | 0.3817971 |
| KEGG_MATURITY_ONSET_DIABETES_OF_THE_YOUNG | 0.38209346 | 1.1608032 | 0.25450903 | 0.3837491 |
| KEGG_ENDOCYTOSIS | 0.2842733 | 1.1580805 | 0.24657534 | 0.3781276 |
| KEGG_PENTOSE_PHOSPHATE_PATHWAY | 0.3725432 | 1.1400403 | 0.3125 | 0.39391986 |
| KEGG_SPHINGOLIPID_METABOLISM | 0.33208936 | 1.1365676 | 0.2896679 | 0.38966054 |
| KEGG_TYROSINE_METABOLISM | 0.3311965 | 1.1281532 | 0.2934363 | 0.3933225 |
| KEGG_HOMOLOGOUS_RECOMBINATION | 0.38157126 | 1.1262164 | 0.3067961 | 0.38782194 |
| KEGG_DRUG_METABOLISM_OTHER_ENZYMES | 0.31365153 | 1.1067797 | 0.31411532 | 0.4053152 |
| KEGG_UBIQUITIN_MEDIATED_PROTEOLYSIS | 0.28718928 | 1.1032367 | 0.3358349 | 0.40224692 |
| KEGG_BLADDER_CANCER | 0.30436495 | 1.0779539 | 0.33950618 | 0.42607406 |
| KEGG_GLYCOLYSIS_GLUCONEOGENESIS | 0.2944187 | 1.0616581 | 0.35521236 | 0.4398082 |
| KEGG_ASCORBATE_AND_ALDARATE_METABOLISM | 0.3758607 | 1.0378919 | 0.42270058 | 0.46389922 |
| KEGG_AMYOTROPHIC_LATERAL_SCLEROSIS_ALS | 0.2707153 | 1.0153503 | 0.42105263 | 0.487548 |
| KEGG_FATTY_ACID_METABOLISM | 0.3012784 | 1.0114101 | 0.42857143 | 0.48468944 |
| KEGG_TRYPTOPHAN_METABOLISM | 0.2972774 | 0.9821446 | 0.4834308 | 0.5185659 |
| KEGG_RNA_DEGRADATION | 0.28542742 | 0.96859914 | 0.48702595 | 0.5291315 |
| KEGG_PENTOSE_AND_GLUCURONATE_INTERCONVERSIONS | 0.3336539 | 0.95896006 | 0.51632655 | 0.5348537 |
| KEGG_HISTIDINE_METABOLISM | 0.2823393 | 0.9298967 | 0.5519231 | 0.56990767 |
| KEGG_ALPHA_LINOLENIC_ACID_METABOLISM | 0.30011782 | 0.9276837 | 0.54563105 | 0.5638387 |
| KEGG_METABOLISM_OF_XENOBIOTICS_BY_CYTOCHROME_P450 | 0.23788722 | 0.8350839 | 0.7057613 | 0.6987177 |
| KEGG_STEROID_HORMONE_BIOSYNTHESIS | 0.25352603 | 0.7880792 | 0.7739307 | 0.7610755 |
| KEGG_RIBOSOME | 0.33705327 | 0.75735134 | 0.6618257 | 0.79585636 |
| KEGG_GLYCINE_SERINE_AND_THREONINE_METABOLISM | 0.2234353 | 0.74398077 | 0.8252427 | 0.8031706 |
| KEGG_REGULATION_OF_AUTOPHAGY | 0.21196257 | 0.6631804 | 0.8944223 | 0.8939575 |
| KEGG_STARCH_AND_SUCROSE_METABOLISM | 0.19122188 | 0.65059894 | 0.934236 | 0.89392644 |
| KEGG_GLYCOSAMINOGLYCAN_DEGRADATION | 0.21291351 | 0.6233715 | 0.8878327 | 0.9072102 |
| KEGG_LINOLEIC_ACID_METABOLISM | 0.18211767 | 0.61535436 | 0.9404762 | 0.9013528 |

NES: normalized enrichment score; NOM: nominal; FDR: false discovery rate. Gene sets with NOM p-val<0.05 and FDR q-val<0.25 are considered as significant.
