# Supplementary material for: CLIP4 Shows Putative Tumor Suppressor Characteristics in Breast Cancer: An Integrated Analysis
Source: Front Mol Biosci. 2021 Jan 26;7:616190. doi: 10.3389/fmolb.2020.616190 (PMC7870488; doi:10.3389/fmolb.2020.616190)
Supplement: Supplementary file 4 [file table4.docx]

**Table S4 Gene sets enriched in phenotype high CLIP4 expression**

| **NAME** | **ES** | **NES** | **NOM p-val** | **FDR q-val** |
| --- | --- | --- | --- | --- |
| KEGG_ARRHYTHMOGENIC_RIGHT_VENTRICULAR_CARDIOMYOPATHY_ARVC | -0.5722377 | -2.1121433 | 0 | 0.057013586 |
| KEGG_BASAL_CELL_CARCINOMA | -0.56872773 | -2.098546 | 0.004357298 | 0.0355268 |
| KEGG_HEDGEHOG_SIGNALING_PATHWAY | -0.52781916 | -2.0100694 | 0 | 0.056891233 |
| KEGG_JAK_STAT_SIGNALING_PATHWAY | -0.50424296 | -1.9909054 | 0.006147541 | 0.051837612 |
| KEGG_FOCAL_ADHESION | -0.5340608 | -1.9848518 | 0.008130081 | 0.04330808 |
| KEGG_PATHWAYS_IN_CANCER | -0.4418863 | -1.9576857 | 0.006097561 | 0.044402122 |
| KEGG_VASCULAR_SMOOTH_MUSCLE_CONTRACTION | -0.46110567 | -1.9497344 | 0.004219409 | 0.04106621 |
| KEGG_ETHER_LIPID_METABOLISM | -0.5305035 | -1.9076972 | 0.006048387 | 0.05143393 |
| KEGG_LEUKOCYTE_TRANSENDOTHELIAL_MIGRATION | -0.5064573 | -1.8914616 | 0.010121457 | 0.053632576 |
| KEGG_CELL_ADHESION_MOLECULES_CAMS | -0.5613885 | -1.8888221 | 0.018480493 | 0.049474813 |
| KEGG_ECM_RECEPTOR_INTERACTION | -0.5850225 | -1.8716356 | 0.016032064 | 0.05304667 |
| KEGG_CALCIUM_SIGNALING_PATHWAY | -0.43547815 | -1.8689628 | 0.002150538 | 0.049678005 |
| KEGG_VIRAL_MYOCARDITIS | -0.57843494 | -1.8603297 | 0.020618556 | 0.049195033 |
| KEGG_AXON_GUIDANCE | -0.4603793 | -1.8585842 | 0.006437768 | 0.04612309 |
| KEGG_REGULATION_OF_ACTIN_CYTOSKELETON | -0.45829087 | -1.8577112 | 0.004201681 | 0.043464288 |
| KEGG_ERBB_SIGNALING_PATHWAY | -0.4630016 | -1.822039 | 0.004282655 | 0.0527396 |
| KEGG_SMALL_CELL_LUNG_CANCER | -0.47366002 | -1.8209468 | 0.012847966 | 0.049927033 |
| KEGG_DORSO_VENTRAL_AXIS_FORMATION | -0.5797559 | -1.8146602 | 0.008695652 | 0.04977964 |
| KEGG_CYTOKINE_CYTOKINE_RECEPTOR_INTERACTION | -0.48612213 | -1.7873968 | 0.02008032 | 0.058052335 |
| KEGG_HYPERTROPHIC_CARDIOMYOPATHY_HCM | -0.49946132 | -1.7769015 | 0.014861995 | 0.059976242 |
| KEGG_DILATED_CARDIOMYOPATHY | -0.48235735 | -1.7417921 | 0.012875536 | 0.07079987 |
| KEGG_WNT_SIGNALING_PATHWAY | -0.3956768 | -1.7230192 | 0.015250545 | 0.076743506 |
| KEGG_TOLL_LIKE_RECEPTOR_SIGNALING_PATHWAY | -0.4591073 | -1.718167 | 0.026422765 | 0.07541847 |
| KEGG_TIGHT_JUNCTION | -0.42362526 | -1.7143764 | 0.012793177 | 0.0742584 |
| KEGG_CHRONIC_MYELOID_LEUKEMIA | -0.44763798 | -1.7110263 | 0.017167382 | 0.07307644 |
| KEGG_NOD_LIKE_RECEPTOR_SIGNALING_PATHWAY | -0.5012893 | -1.7084852 | 0.032323234 | 0.071320646 |
| KEGG_MELANOGENESIS | -0.41483817 | -1.7060171 | 0.01268499 | 0.06974019 |
| KEGG_MAPK_SIGNALING_PATHWAY | -0.38080686 | -1.7012707 | 0.016842104 | 0.069468245 |
| KEGG_GLYCOSAMINOGLYCAN_BIOSYNTHESIS_CHONDROITIN_SULFATE | -0.5985287 | -1.6919262 | 0.0332681 | 0.07149642 |
| KEGG_ADHERENS_JUNCTION | -0.45983106 | -1.686354 | 0.023655914 | 0.07201142 |
| KEGG_B_CELL_RECEPTOR_SIGNALING_PATHWAY | -0.5015318 | -1.6841147 | 0.045833334 | 0.070646815 |
| KEGG_GLYCOSAMINOGLYCAN_BIOSYNTHESIS_HEPARAN_SULFATE | -0.5128079 | -1.683134 | 0.018292682 | 0.06889961 |
| KEGG_ACUTE_MYELOID_LEUKEMIA | -0.45153803 | -1.6788578 | 0.021097047 | 0.06877734 |
| KEGG_NEUROACTIVE_LIGAND_RECEPTOR_INTERACTION | -0.3706717 | -1.6676286 | 0.002066116 | 0.071248636 |
| KEGG_T_CELL_RECEPTOR_SIGNALING_PATHWAY | -0.48614466 | -1.6640894 | 0.054968286 | 0.07074104 |
| KEGG_TGF_BETA_SIGNALING_PATHWAY | -0.43216255 | -1.6396987 | 0.042424243 | 0.07988503 |
| KEGG_FC_GAMMA_R_MEDIATED_PHAGOCYTOSIS | -0.43489954 | -1.6291196 | 0.045360826 | 0.08314109 |
| KEGG_COLORECTAL_CANCER | -0.42979583 | -1.6268406 | 0.040339705 | 0.08186343 |
| KEGG_APOPTOSIS | -0.42107514 | -1.6200927 | 0.050955415 | 0.08303626 |
| KEGG_GLYCEROPHOSPHOLIPID_METABOLISM | -0.39324817 | -1.6166506 | 0.014373717 | 0.082854845 |
| KEGG_GAP_JUNCTION | -0.41107172 | -1.6042249 | 0.026639344 | 0.08687467 |
| KEGG_GLYCOSPHINGOLIPID_BIOSYNTHESIS_GANGLIO_SERIES | -0.577263 | -1.6003838 | 0.03265306 | 0.0868249 |
| KEGG_CHEMOKINE_SIGNALING_PATHWAY | -0.4251156 | -1.5942006 | 0.07786885 | 0.08778774 |
| KEGG_GLIOMA | -0.41528425 | -1.5941174 | 0.024793388 | 0.08583158 |
| KEGG_ADIPOCYTOKINE_SIGNALING_PATHWAY | -0.4164541 | -1.5748988 | 0.0373444 | 0.09366444 |
| KEGG_PHOSPHATIDYLINOSITOL_SIGNALING_SYSTEM | -0.40937787 | -1.5730512 | 0.061965812 | 0.09255173 |
| KEGG_RENAL_CELL_CARCINOMA | -0.4074751 | -1.5726093 | 0.041666668 | 0.09076186 |
| KEGG_INTESTINAL_IMMUNE_NETWORK_FOR_IGA_PRODUCTION | -0.59215033 | -1.5694137 | 0.11290322 | 0.090670206 |
| KEGG_LEISHMANIA_INFECTION | -0.5193456 | -1.5614294 | 0.11422846 | 0.093096435 |
| KEGG_SYSTEMIC_LUPUS_ERYTHEMATOSUS | -0.5636549 | -1.5521128 | 0.10728745 | 0.09571057 |
| KEGG_GLYCOSPHINGOLIPID_BIOSYNTHESIS_LACTO_AND_NEOLACTO_SERIES | -0.4927907 | -1.5506274 | 0.05421687 | 0.094475776 |
| KEGG_NON_SMALL_CELL_LUNG_CANCER | -0.42283732 | -1.545913 | 0.044871796 | 0.09523668 |
| KEGG_ABC_TRANSPORTERS | -0.43721253 | -1.5344464 | 0.057142857 | 0.098448195 |
| KEGG_HEMATOPOIETIC_CELL_LINEAGE | -0.48827824 | -1.5255647 | 0.114583336 | 0.101014756 |
| KEGG_ALDOSTERONE_REGULATED_SODIUM_REABSORPTION | -0.44005895 | -1.5178186 | 0.04535637 | 0.10287762 |
| KEGG_TASTE_TRANSDUCTION | -0.42453584 | -1.5152366 | 0.036809817 | 0.1024051 |
| KEGG_NATURAL_KILLER_CELL_MEDIATED_CYTOTOXICITY | -0.42755133 | -1.5135024 | 0.11485148 | 0.10156405 |
| KEGG_MELANOMA | -0.38152567 | -1.5028359 | 0.034274194 | 0.10551583 |
| KEGG_GNRH_SIGNALING_PATHWAY | -0.3497614 | -1.482813 | 0.04525862 | 0.11534601 |
| KEGG_PROGESTERONE_MEDIATED_OOCYTE_MATURATION | -0.38385934 | -1.4804205 | 0.06924643 | 0.11466614 |
| KEGG_ASTHMA | -0.56942105 | -1.4694996 | 0.16194332 | 0.11913701 |
| KEGG_PANCREATIC_CANCER | -0.37961254 | -1.4646522 | 0.07531381 | 0.11976856 |
| KEGG_LONG_TERM_POTENTIATION | -0.35889104 | -1.4615142 | 0.0770878 | 0.11996723 |
| KEGG_PRION_DISEASES | -0.48654073 | -1.4430473 | 0.09406953 | 0.12894706 |
| KEGG_FC_EPSILON_RI_SIGNALING_PATHWAY | -0.37220812 | -1.4420004 | 0.083690986 | 0.12742376 |
| KEGG_MTOR_SIGNALING_PATHWAY | -0.3803225 | -1.4307382 | 0.07114624 | 0.1324071 |
| KEGG_GLYCEROLIPID_METABOLISM | -0.37812066 | -1.4217073 | 0.056565657 | 0.13616672 |
| KEGG_P53_SIGNALING_PATHWAY | -0.36708385 | -1.402219 | 0.1010101 | 0.14681664 |
| KEGG_PROSTATE_CANCER | -0.36279613 | -1.3976938 | 0.089361705 | 0.14716613 |
| KEGG_NEUROTROPHIN_SIGNALING_PATHWAY | -0.33813375 | -1.3902769 | 0.11087866 | 0.14994545 |
| KEGG_PATHOGENIC_ESCHERICHIA_COLI_INFECTION | -0.41015893 | -1.3691679 | 0.12301587 | 0.16275457 |
| KEGG_AUTOIMMUNE_THYROID_DISEASE | -0.48986113 | -1.3579721 | 0.18164794 | 0.16886808 |
| KEGG_PRIMARY_IMMUNODEFICIENCY | -0.564351 | -1.3535963 | 0.22129436 | 0.16971251 |
| KEGG_THYROID_CANCER | -0.39479533 | -1.3391962 | 0.12715517 | 0.17838098 |
| KEGG_CYSTEINE_AND_METHIONINE_METABOLISM | -0.38861102 | -1.3316668 | 0.13861386 | 0.18167853 |
| KEGG_VEGF_SIGNALING_PATHWAY | -0.3336503 | -1.3276223 | 0.09850107 | 0.18233837 |
| KEGG_ALLOGRAFT_REJECTION | -0.5641844 | -1.3022125 | 0.27237353 | 0.19999665 |
| KEGG_INOSITOL_PHOSPHATE_METABOLISM | -0.35168365 | -1.2920552 | 0.17975207 | 0.20531201 |
| KEGG_GRAFT_VERSUS_HOST_DISEASE | -0.54386955 | -1.2908858 | 0.29366603 | 0.20366634 |
| KEGG_INSULIN_SIGNALING_PATHWAY | -0.30226249 | -1.2640307 | 0.1431624 | 0.22391681 |
| KEGG_LONG_TERM_DEPRESSION | -0.31187326 | -1.2554016 | 0.17047817 | 0.22921874 |
| KEGG_ENDOMETRIAL_CANCER | -0.3407907 | -1.2495071 | 0.18461539 | 0.2311767 |
| KEGG_NOTCH_SIGNALING_PATHWAY | -0.35551044 | -1.2073059 | 0.2360515 | 0.2678068 |
| KEGG_OOCYTE_MEIOSIS | -0.30398074 | -1.1705513 | 0.26987448 | 0.3035517 |
| KEGG_GLYCOSAMINOGLYCAN_BIOSYNTHESIS_KERATAN_SULFATE | -0.42217752 | -1.1616951 | 0.28225806 | 0.31010175 |
| KEGG_TYPE_II_DIABETES_MELLITUS | -0.30813995 | -1.1410655 | 0.2699115 | 0.32973382 |
| KEGG_TYPE_I_DIABETES_MELLITUS | -0.427416 | -1.1385036 | 0.3653484 | 0.32863307 |
| KEGG_EPITHELIAL_CELL_SIGNALING_IN_HELICOBACTER_PYLORI_INFECTION | -0.29469445 | -1.1216143 | 0.30816326 | 0.34366328 |
| KEGG_PPAR_SIGNALING_PATHWAY | -0.31959993 | -1.093167 | 0.32329318 | 0.37213615 |
| KEGG_COMPLEMENT_AND_COAGULATION_CASCADES | -0.4204787 | -1.0641924 | 0.3952569 | 0.40530407 |
| KEGG_PRIMARY_BILE_ACID_BIOSYNTHESIS | -0.36804616 | -1.0584625 | 0.38181818 | 0.40759197 |
| KEGG_RIG_I_LIKE_RECEPTOR_SIGNALING_PATHWAY | -0.27156025 | -1.0172005 | 0.4321503 | 0.45641935 |
| KEGG_NITROGEN_METABOLISM | -0.31186268 | -1.0078005 | 0.42768595 | 0.4642558 |
| KEGG_ARACHIDONIC_ACID_METABOLISM | -0.27208355 | -0.9812205 | 0.4626556 | 0.49653092 |
| KEGG_GALACTOSE_METABOLISM | -0.31815737 | -0.9779045 | 0.4597938 | 0.49604747 |
| KEGG_RENIN_ANGIOTENSIN_SYSTEM | -0.32433838 | -0.9776655 | 0.5 | 0.49124685 |
| KEGG_ONE_CARBON_POOL_BY_FOLATE | -0.33117592 | -0.96069044 | 0.49685535 | 0.5102809 |
| KEGG_O_GLYCAN_BIOSYNTHESIS | -0.28927705 | -0.95254713 | 0.5142276 | 0.51675767 |
| KEGG_PROXIMAL_TUBULE_BICARBONATE_RECLAMATION | -0.30276045 | -0.951907 | 0.5092025 | 0.5123913 |
| KEGG_RETINOL_METABOLISM | -0.25433743 | -0.92475003 | 0.56777996 | 0.5469807 |
| KEGG_CYTOSOLIC_DNA_SENSING_PATHWAY | -0.2674111 | -0.9195789 | 0.5612648 | 0.5491206 |
| KEGG_CELL_CYCLE | -0.26592678 | -0.9064785 | 0.53752536 | 0.5625454 |
| KEGG_LYSINE_DEGRADATION | -0.25988552 | -0.9018583 | 0.56008583 | 0.56387115 |
| KEGG_BASAL_TRANSCRIPTION_FACTORS | -0.27079147 | -0.88583785 | 0.5758197 | 0.58170545 |
| KEGG_NICOTINATE_AND_NICOTINAMIDE_METABOLISM | -0.26490623 | -0.86724144 | 0.6464435 | 0.6028559 |
| KEGG_DRUG_METABOLISM_CYTOCHROME_P450 | -0.23190673 | -0.8645273 | 0.6607495 | 0.6009217 |
| KEGG_BETA_ALANINE_METABOLISM | -0.26349297 | -0.8558237 | 0.6581197 | 0.6072457 |
| KEGG_OLFACTORY_TRANSDUCTION | -0.28807688 | -0.79315025 | 0.8162476 | 0.69409 |
| KEGG_ANTIGEN_PROCESSING_AND_PRESENTATION | -0.22431844 | -0.6999266 | 0.74088293 | 0.8149403 |

NES: normalized enrichment score; NOM: nominal; FDR: false discovery rate. Gene sets with NOM p-val<0.05 and FDR q-val<0.25 are considered as significant.
