# Supplementary material for: CLIP4 Shows Putative Tumor Suppressor Characteristics in Breast Cancer: An Integrated Analysis
Source: Front Mol Biosci. 2021 Jan 26;7:616190. doi: 10.3389/fmolb.2020.616190 (PMC7870488; doi:10.3389/fmolb.2020.616190)
Supplement: Supplementary file 5 [file image1.pdf]

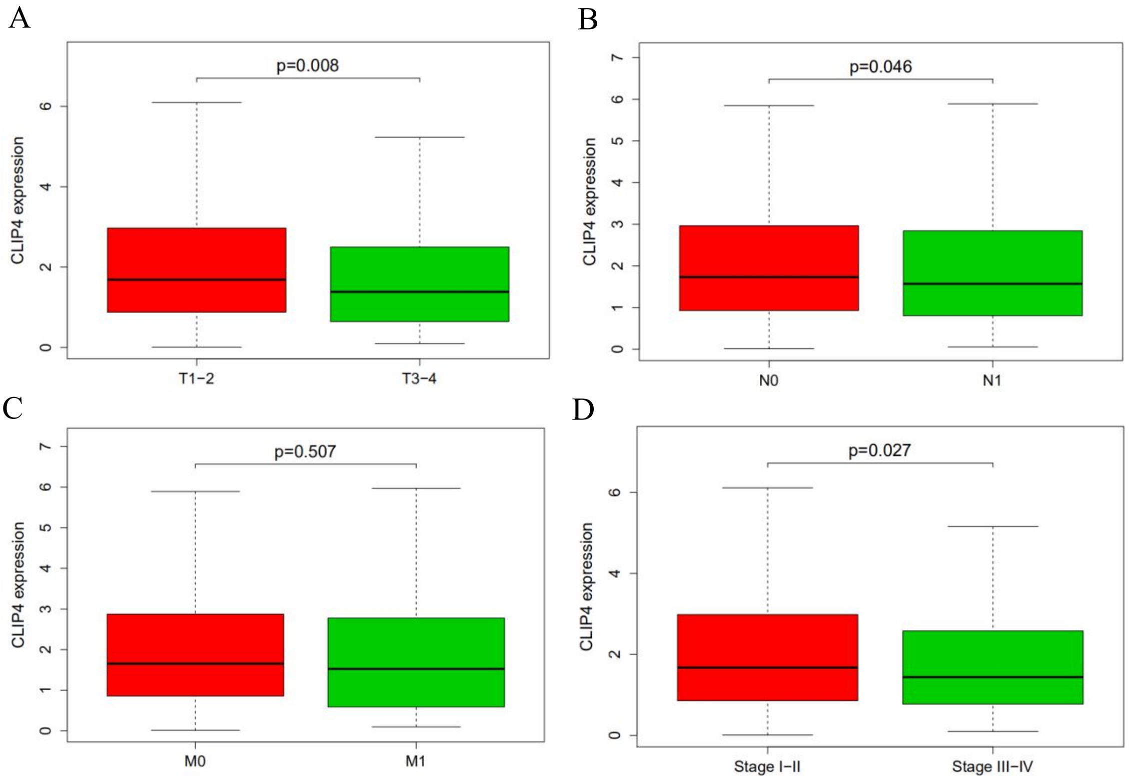

Figure S1 The associations between CLIP4 expression and TNM stage in patients with breast cancer based on TCGA datasets. (A) Boxplot of CLIP4 expression according to T (T1-2 vs. T3-4); (B) Boxplot of CLIP4 expression according to N (N0 vs. N1); (C) Boxplot of CLIP4 expression according to M (M0 vs. M1); (D) Boxplot of CLIP4 expression according to stage (stage I-II vs. stage III-IV).
