# Supplementary material for: CLIP4 Shows Putative Tumor Suppressor Characteristics in Breast Cancer: An Integrated Analysis
Source: Front Mol Biosci. 2021 Jan 26;7:616190. doi: 10.3389/fmolb.2020.616190 (PMC7870488; doi:10.3389/fmolb.2020.616190)
Supplement: Supplementary file 6 [file image2.pdf]

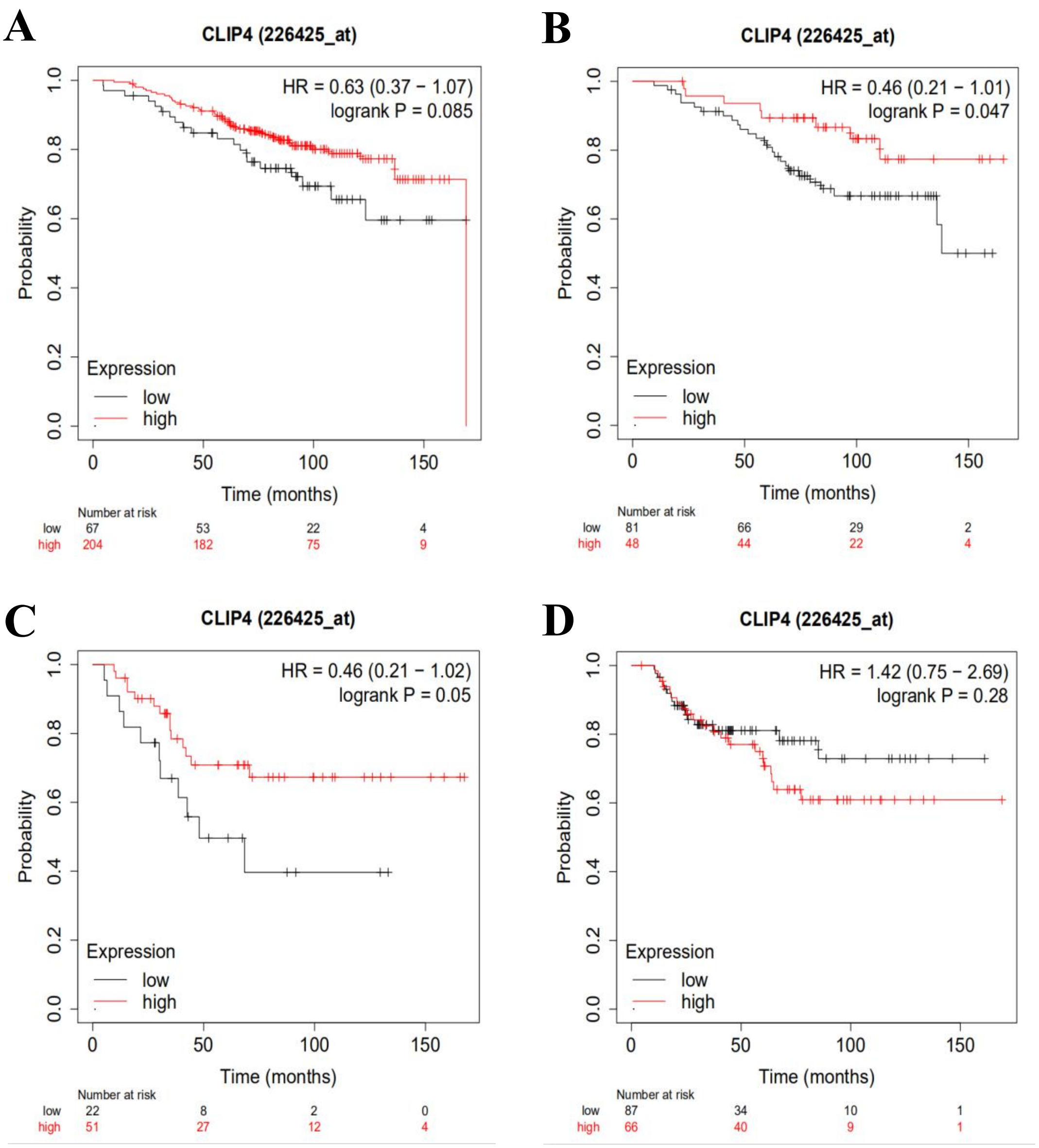

Figure S2 The prognostic value for CLIP4 in different intrinsic subtypes of breast cancer. (A) High expression of CLIP4 indicates a better OS in luminal A breast cancer (p=0.085, Kaplan-Meier Plotter datasets); (B) High expression of CLIP4 indicates a better OS in luminal B breast cancer (p=0.047, Kaplan-Meier Plotter datasets); (C) High expression of CLIP4 indicates a better OS in HER2 positive breast cancer (p=0.050, Kaplan-Meier Plotter datasets); (D) High expression of CLIP4 indicates a poor OS in basal-like breast cancer (p=0.280, Kaplan-Meier Plotter datasets).
