# Supplementary material for: CLIP4 Shows Putative Tumor Suppressor Characteristics in Breast Cancer: An Integrated Analysis
Source: Front Mol Biosci. 2021 Jan 26;7:616190. doi: 10.3389/fmolb.2020.616190 (PMC7870488; doi:10.3389/fmolb.2020.616190)
Supplement: Supplementary file 8 [file image4.pdf]

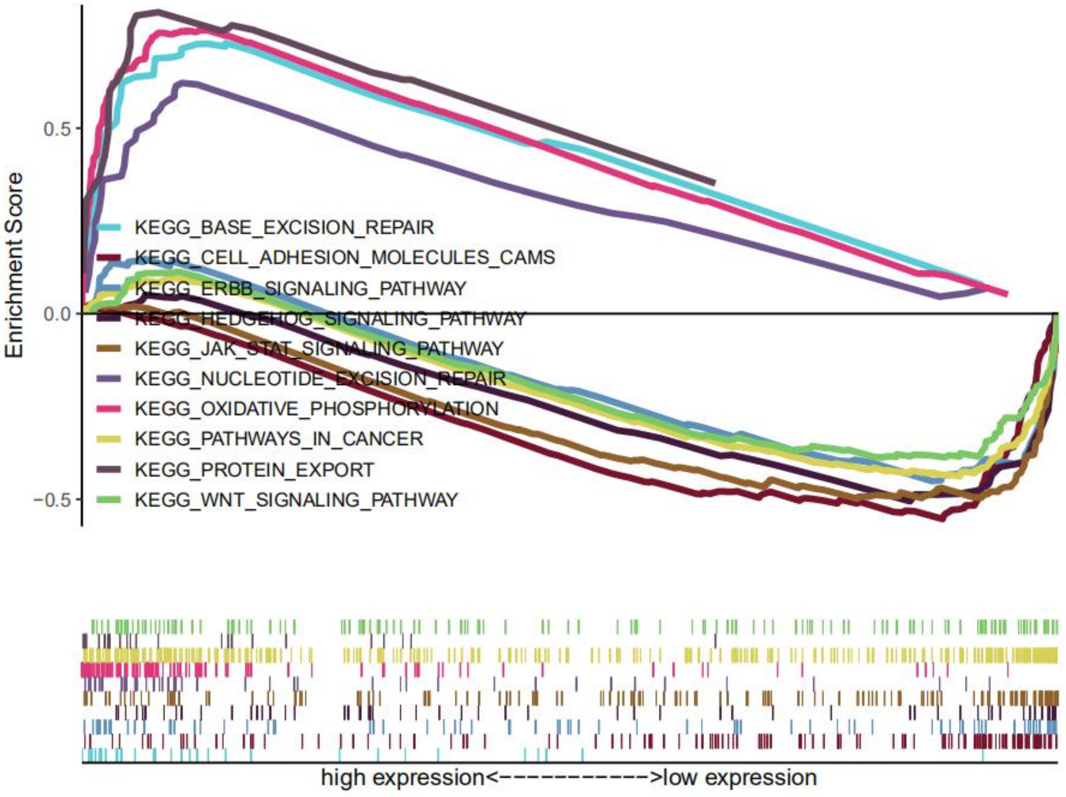

Figure S4 Enrichment plots for CLIP4 in breast cancer obtained by multiple GSEA. GSEA results showing Wnt signaling pathway etc. are differentially enriched in CLIP4-high and -low expression phenotype.
