# Supplementary material for: CLIP4 Shows Putative Tumor Suppressor Characteristics in Breast Cancer: An Integrated Analysis
Source: Front Mol Biosci. 2021 Jan 26;7:616190. doi: 10.3389/fmolb.2020.616190 (PMC7870488; doi:10.3389/fmolb.2020.616190)
Supplement: Supplementary file 9 [file image5.pdf]

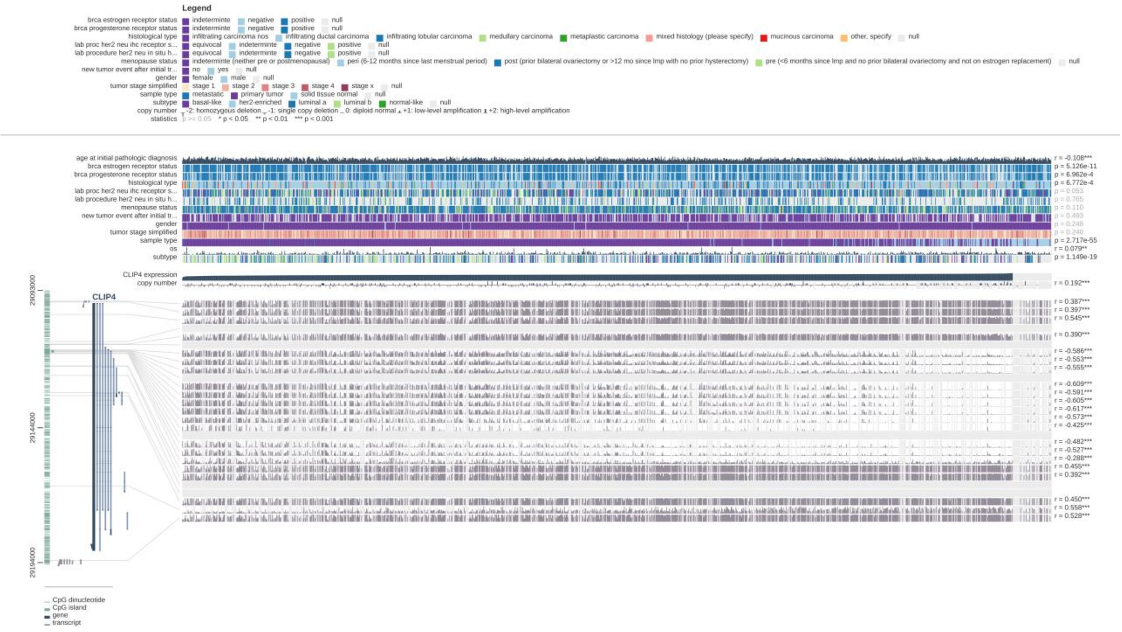

Figure S5 Visualization of DNA methylation and mRNA expression data for CLIP4 in breast cancer analyzed by MEXPRESS. 871 breast invasive carcinoma samples were included. DNA methylation data for each probe were determined using Infinium Human Methylation 450 microarray. Gene expression data were derived from Illumina HiSeq RNASeqV2. The samples are ascendingly ordered by the level of expression. The significance of the relation (correlation coefficient r or p value) between each row of data (clinical data, expression or methylation) was shown in the right side. *p<0.05; **p<0.01; ***p<0.001.
